# Cytoplasmic strings between ICM and mTE are a positive predictor of clinical pregnancy and live birth outcomes: A time-lapse study

**DOI:** 10.3389/fmed.2022.934327

**Published:** 2022-07-28

**Authors:** Bing-Xin Ma, Liu Yang, Yu Tian, Lei Jin, Bo Huang

**Affiliations:** ^1^Reproductive Medicine Center, Tongji Hospital, Tongji Medical College, Huazhong University of Science and Technology, Wuhan, China; ^2^Jiangsu Hengrui Pharmaceuticals Co., Ltd., Shanghai, China

**Keywords:** cytoplasmic string, elective single blastocyst transfer, inner cell mass, trophectoderm, time lapse

## Abstract

**Background:**

Elective single blastocyst transfer (eSBT) is considered to reduce the incidence of multiple pregnancy compared to double embryo transfer. Blastocyst selection is the key to achieving pregnancy. In the past, morphological assessment was the main criterion used to select blastocyst. Some important morphological parameters are considered to be clinically valuable, such as cytoplasmic strings traversing from the inner cell mass (ICM) and mural trophectoderm (mTE).

**Methods:**

In this study, 1,267 elective frozen-thawed eSBT cycles cultured in a time-lapse culture system from January 2018 to May 2019 were included. Blastocysts were grouped into “present” and “absent” according to the appearance of cytoplasmic strings between ICM and mTE cells. The “present” group was further categorized according to the quantity of cytoplasmic strings between the ICM and mTE cells.

**Results:**

A time-lapse analysis indicated that cytoplasmic strings between ICM and mTE were more visible among good quality blastocysts. Furthermore, blastocysts with cytoplasmic strings showed higher clinical pregnancy and live birth rates (*P* = 0.011 and 0.003), while no significant differences were observed in abortion rate and birth weight (*P* = 0.466 and 0.556).

**Conclusions:**

In conclusion, although the results of previous studies about cytoplasmic strings have been controversial, the present time-lapse analysis provides evidence for the first time that cytoplasmic strings between ICM and mTE cells are a positive predictor of clinical pregnancy and live birth outcomes in elective frozen-thawed single blastocyst transfer cycles.

## Introduction

The goal of assisted reproductive technology is to achieve a healthy singleton birth and minimize maternal and neonatal risk (1–3). In order to realize this goal, elective single blastocyst transfer (eSBT) has been preferred in recent decades (4, 5). Blastocyst transfer is considered physiologically appropriate because it more closely mimics the time of natural implantation and may improve synchrony between the endometrium and embryo development (6, 7). It has been reported that the clinical pregnancy and live birth rates resulting from elective single blastocyst transfer (eSBT) are similar to those of double embryo transfer, and higher than those of single cleavage-stage embryo transfer. Furthermore, eSBT generates a reduced incidence of multiple pregnancy compared to double embryo transfer (8–10).

With the development of cryopreservation technology, more embryos have been cryopreserved for further use. Some studies have reported that frozen-thawed embryo transfer could lead to a higher live birth rate in women with polycystic ovary syndrome. Frozen-thawed embryo transfer could also avoid exposure of the endometrium to the adverse sequelae of ovarian stimulation (11). Additionally, a supraphysiologic hormonal milieu increases the incidence of ectopic pregnancy in fresh cycles. Thus, the frozen-thawed eSBT strategy can not only optimize pregnancy rates, but also maintain perinatal safety (12). Blastocyst selection is the key to a successful eSBT, as it is important to shorten the time to pregnancy (13). In the past, morphological assessment was the main criterion used to select blastocysts. The blastocyst grading system by Gardner and Schoolcraft remains largely unchallenged and is widely used in the clinic (14). However, this system does not include some important morphological parameters often observed in the *in vitro* fertilization (IVF) laboratory. Albeit of unknown importance, some of these parameters were briefly introduced in the Istanbul consensus document, e.g., the formation of cytoplasmic strings that are often found linking together different cells and cell types (15).

Methods for embryo selection have greatly improved over the past decade, including not only static morphological assessment, but also integration of non-invasive time-lapse analysis. Cytoplasmic strings can be observed between the oocyte and zona pellucida (ZP), the outside surface of blastomere, and traversing from the inner cell mass (ICM) and mural trophectoderm (mTE) with use of a time-lapse system. It has been suggested that these strings allow a selective and bidirectional communication between mitotic mTE and ICM cells (16). However, the impact of cytoplasmic strings on the clinical outcome for a blastocyst remains controversial (17).

In this study, we aimed to determine whether cytoplasmic strings between ICM and mTE cells are a positive predictor of clinical pregnancy and live birth in frozen-thawed eSBT cycles.

## Materials and methods

### Study design

**Figure 1:**
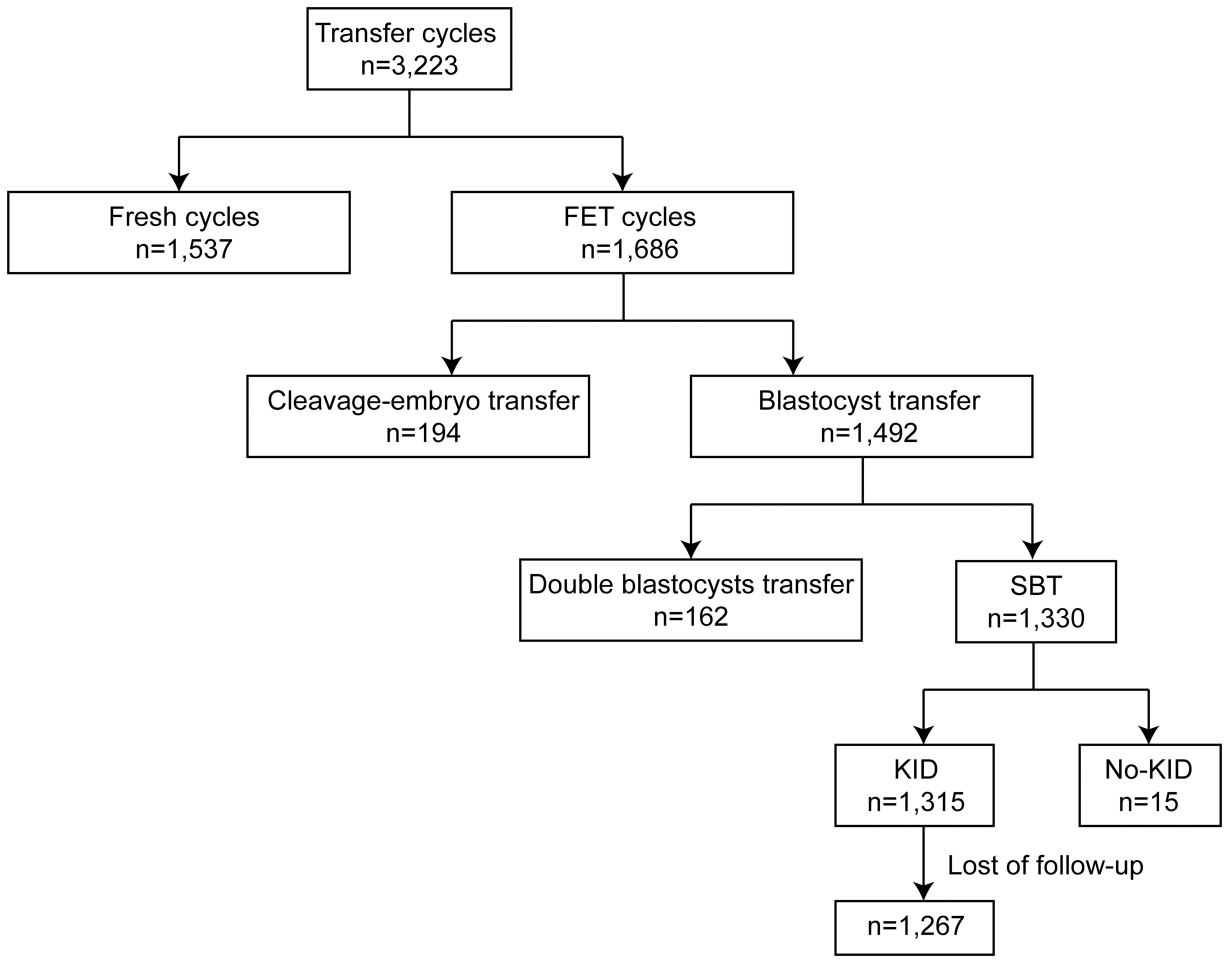
Schematic presentation of the study design. SBT, single blastocyst transfer; KID, known implantation data. FET, frozen embryo transfer[[Inline Image]].

In the present study, we retrospectively analyzed 3,223 transfer cycles with a time-lapse incubation system (EmbryoScope Plus, Vitrolife, Sweden) from January 2018 to May 2019 at the Reproductive Medicine Center, Tongji Hospital, Tongji Medical College, Huazhong University of Science and Technology, Wuhan, Hubei, China. 1,537 fresh cycles and 1,686 frozen embryo transfer (FET) cycles were initially included (Figure 1). Among FET cycles, 194 cleavage embryos and 1,492 blastocysts were transferred. Across all blastocyst transfers, there were 1,330 eSBT cycles. After exclusion of no-known implantation data (KID) eSBT cycles and loss of follow-up, 1,267 eSBT cycles were included in the final study. All patients gave written informed consent. The study conformed to the Declaration of Helsinki for Medical Research involving human subjects. Institutional review board (No. 2019s097) approval was given by the Ethical Committee of Reproductive Medicine Center, Tongji Hospital, Tongji Medicine College, Huazhong University of Science and Technology.

### Clinical protocol

Controlled ovarian stimulation (COS) was performed on patients as per our previous paper (18, 19). During COS, patients were monitored closely using transvaginal ultrasound. Human chorionic gonadotropin (HCG) was given when the leading follicle (s) was >18 mm. Thirty-six hours after HCG injection, ultrasound-guided oocyte retrieval was conducted.

### Laboratory protocol

The protocol of semen/cumulus-oocyte complexes (COCs) preparation, insemination, and embryo culturing was performed as described in our previous paper (20). Density gradient centrifugation was used to optimize the semen samples. The concentration, motility, and morphology of sperm were evaluated according to the fifth edition of World Health Organization (WHO) guidelines. In IVF cycles, each COC was inseminated with 10,000 motile spermatozoa 3–4 h after retrieval. In intracytoplasmic sperm injection (ICSI) cycles, the COCs were denuded 2 h later, and sperm was injected 4 h after retrieval. Resulting zygotes were transferred to G1 Plus (Vitrolife, Sweden) and cultured in a time-lapse incubation system. The system was set to take pictures of each embryo every 10 min. Pronuclei were checked 16–18 h after insemination. On day 3, the media was replaced by G2 Plus (Vitrolife, Sweden). On days 5 and 6, blastocysts were cryopreserved for further use. All culture media used were Vitrolife G-series media (Vitrolife, Sweden).

### FET protocols

The FET protocols followed were those established in previous papers (21). For natural cycles, endometrial thickness, follicle growth, and ovulation were measured by transvaginal ultrasound examination, with measurement of serum progesterone levels from cycle day 10–12. Thawing and transferring of blastocysts was conducted 3 d after ovulation. From 1 d after ovulation, progesterone was injected intramuscularly for luteal support.

For hormone replacement treatment cycles, oral estradiol (Progynova; Bayer; Leverkusen) was given at 2 mg/day from 1 to 4 cycle days, 4 mg/day from days 5 to 8, and 6 mg/day from days 9 to 12. On the 13th day, when the thickness of the endometrium reached 8.0mm or the maximum, 40 mg of progesterone was injected intramuscularly for 3 d. The blastocyst was thawed and transplanted on the 4th day after 3 d of progesterone administration (22).

Four weeks after transfer, fetal cardiac activity in the uterus was observed *via* transvaginal ultrasound to confirm clinical pregnancy. Miscarriage was defined as loss of the gestational sac or fetal heart activity within 20 weeks after confirming clinical pregnancy.

### Grouping

**Figure 2:**
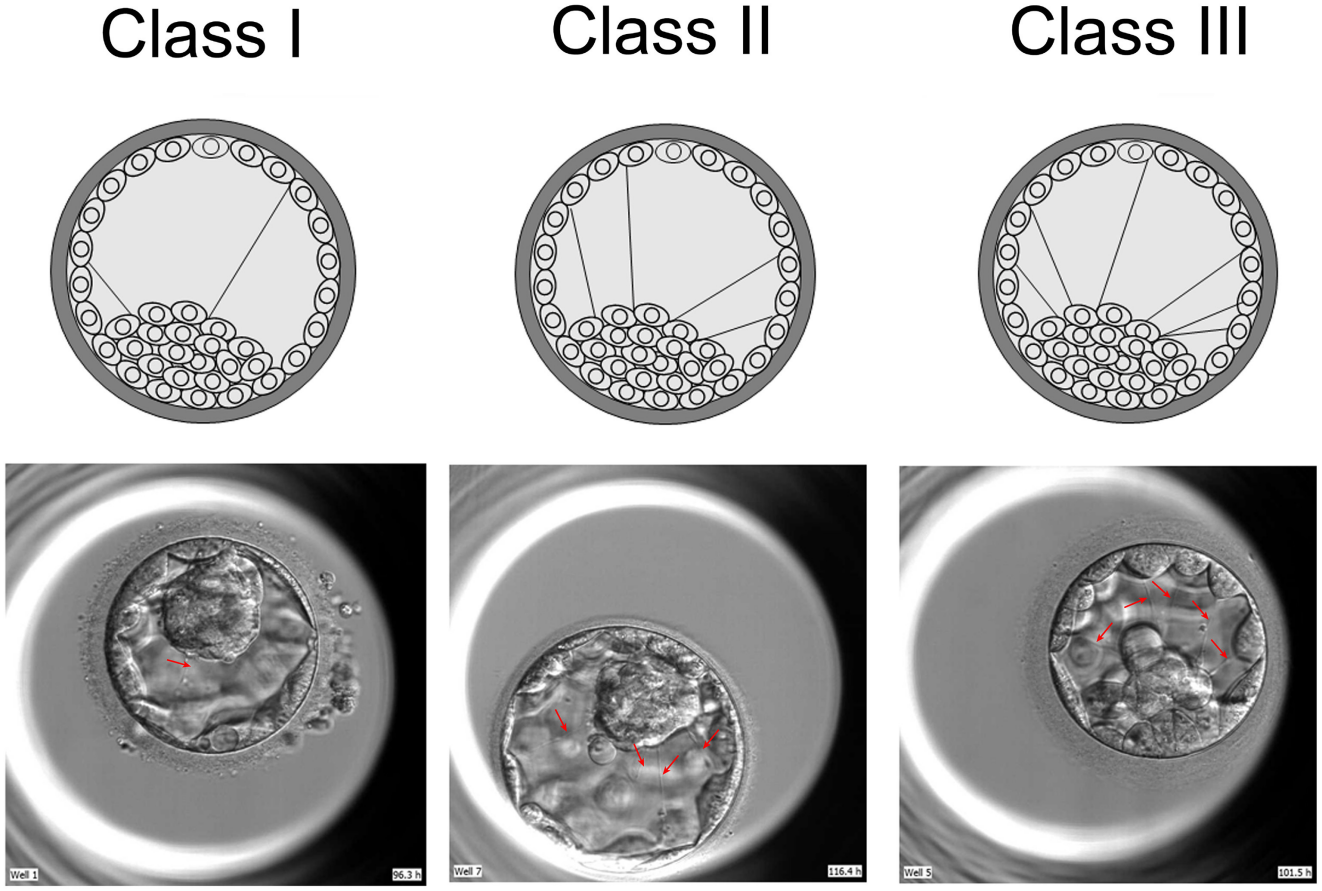
Classification of cytoplasmic strings between ICM and mTE cells. Threads were classified into three groups, based on their quantity: single and double string (s) (I), three to four strings (II) and more than four strings (III). ICM, inner cell mass; mTE, mural trophectoderm.

The blastocysts were photographed every 5 mins by time lapse system. Two embryologists examined all pictures taken of the blastocysts from the emergence of the cavity to vitrification. These examiners were blind to other clinical parameters. Blastocysts were grouped according to the presence (“present”) or absence (“absent”) of cytoplasmic strings between ICM and mTE cells. The “present” group was further categorized according to the quantity of cytoplasmic strings between ICM and mTE cells as follows: one or two strings (Class I); three to four strings (Class II); and five or more strings (Class III) (Figure 2).

### Statistical analysis

Statistical analysis was performed with Statistical Package for Social Sciences (SPSS) version 19.0 (SPSS Inc., USA). Continuous variables with non-normal distributions were expressed as median and interquartile range (IQR). Categorical variables were expressed as number and percentage (%). Differences of continuous variables in baseline characteristics and outcomes were evaluated by Mann-Whitney U test for two groups and Kruskal-Wallis test followed by Dunn's test for multiple comparisons. Chi-square analysis was performed to compare the categorical variables of baseline characteristics and outcomes. Statistical significance was established at *P* < 0.05 level.

## Results

### Patient and cycle characteristics

A total of 1,267 frozen-thawed eSBT cycles at Reproductive Medicine Center, Tongji Hospital, Tongji Medical College, Huazhong University of Science and Technology were involved in the cohort study. 1,002 cycles (79.08%) had observable cytoplasmic strings between ICM and mTE cells and were assigned to the “present” group, and 265 cycles (20.92%) had no observable strings and were assigned to the “absent” group. The cytoplasmic strings were identified on the day of vitrification by time-lapse system. If the cavity of blastocyst collapsed, it was considered by the maximum number of strings it had present.

Patients in the two groups were similar in baseline demographic features. There were no significant differences between the two groups for average maternal age, maternal BMI, ratio of primary subfertility, duration of subfertility, or values for FSH and AMH (Table 1). The cycle characteristics, such as insemination method, oocytes, MII, 2PN, FET protocol, and endometrial thickness were also similar (Table 1).

**Table 1:** Baseline demographic features of the included patients and cycles.

	**Present**	**Absent**	***P***
	**(*n* = 1,002)**	**(*n* = 265)**	
**Demographic parameters**			
Maternal age (y)	31.00 (28.00–34.00)	30.50 (28.00–34.00)	0.730^a^
Maternal BMI	21.26 (19.48–23.16)	21.80 (19.56–23.60)	0.213 ^a^
Duration of subfertility (y)	2.50 (1.00–4.00)	3.00 (2.00–5.00)	0.026 ^a^
Primary subfertility	711/1002 (70.96)	190/265 (71.7)	0.298^b^
**Clinical parameters**			
FSH (IU/L)	7.26 (6.30–8.43)	7.29 (6.22–8.48)	0.897 ^a^
AMH (ng/ml)	5.50 (3.46–8.19)	5.57 (3.35–8.00)	0.905 ^a^
Hormone replacement treatment cycles	977/1,002 (97.50)	258/265 (97.36)	0.721 ^b^
Natural cycles	25/1,002(2.50)	7/265 (2.64)	0.812 ^b^
Endometrial thickness (mm)	11.40 (9.50–13.28)	11.60 (9.45–13.65)	0.781 ^a^
**Treatments**			
IVF Fertilization	582/1,002 (58.08)	158/265 (59.62)	0.414 ^b^
ICSI Fertilization	420/1,002 (41.92)	107/265 (40.38)	0.414 ^b^
**Embryonic parameters**			
Oocytes	13.00 (11.00–16.00)	13.00 (11.00–16.00)	0.233 ^a^
MII	11.00 (9.00–13.00)	11.00 (9.00–13.00)	0.474 ^a^
2PN	8.00 (6.00–8.00)	8.00 (6.00–8.00)	0.116 ^a^

*Data are presented as median (IQR) or number (%)*.

*^a^The P value were calculated by Mann-Whitney U test.*

*^b^The P value were calculated by Chi-square test*.

All blastocysts observed with cytoplasmic strings between ICM and mTE cells (the “present” group) were classified according to the quantity of cytoplasmic strings. 572 blastocysts (57.09%) were allocated to Class I (one or two strings), 366 blastocysts (36.53%) to Class II (three or four strings), and 64 blastocysts (6.39%) to Class III (five or more strings).

### The frequency of strings observed in different blastocyst stages

**Figure 3:**
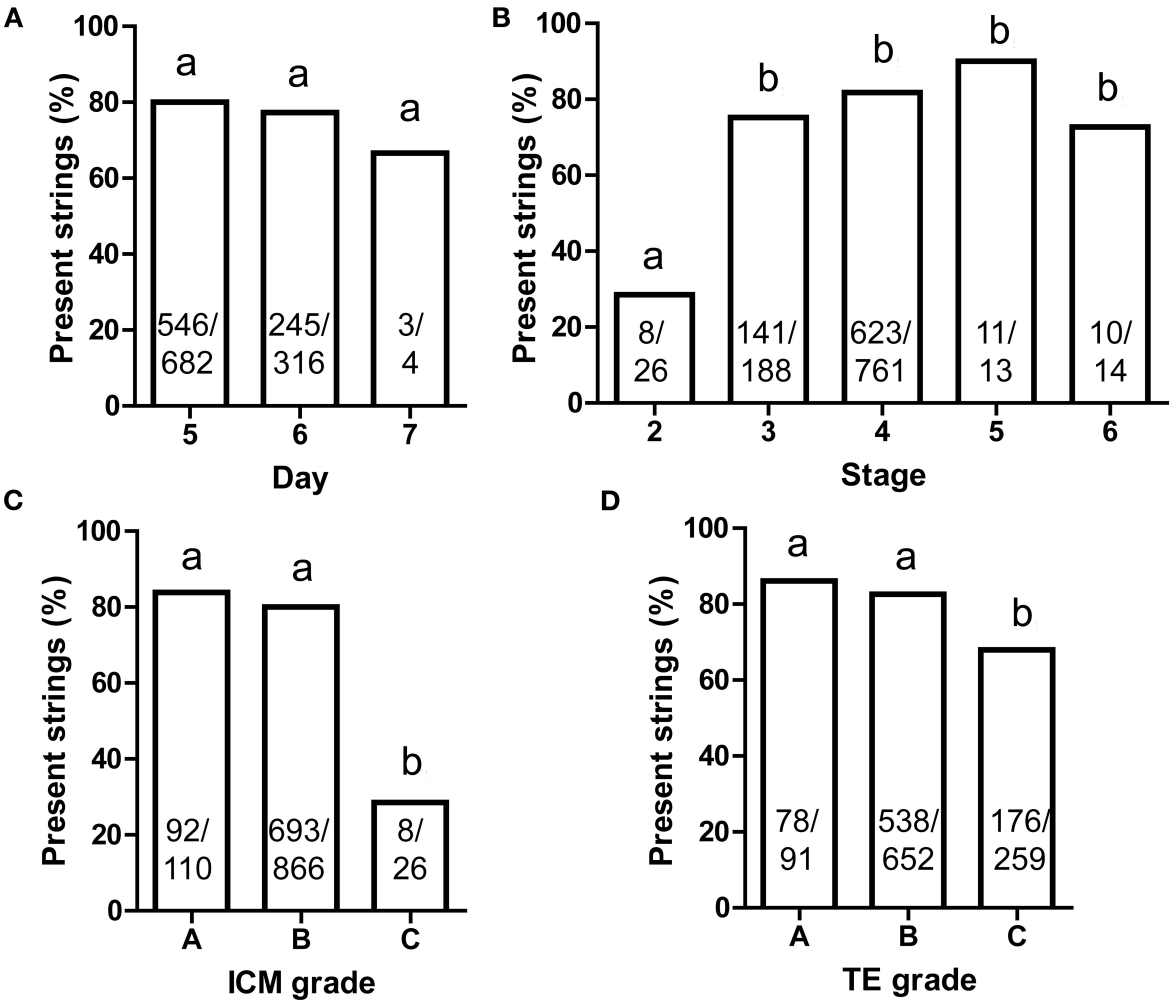
The present frequency of cytoplasmic strings between ICM and mTE cells. **(A)** Different blastocyst ages; **(B)** different blastocyst stages; **(C)** different ICM grades; **(D)** different TE grades. Throughout, multiple Chi-square analysis was performed **(A–D)** and the same superscript lowercase letters indicated no statistical differences in the intergroup comparisons (*P* > 0.05). ICM, inner cell mass; mTE, mural trophectoderm. The sample size was presented on each column.

The blastocyst development stage and quality of ICM and TE were assessed according to the method of David Gardner as follows: Stage 2: the volume of the blastocoel is not less than half of the total volume of the blastocyst; Stage 3: the blastocoel completely occupies the total volume of the blastocyst; Stage 4: the blastocyst expands, the blastocoel completely fills the blastocyst, the total volume of the embryo becomes larger, and the zona pellucida becomes thinner; Stage 5: the hatching blastocyst; Stage 6: the blastocyst hatches from ZP (14). In most cases in the clinic, blastocysts above 3BC level were vitrified and transferred. Most transferred blastocysts were vitrified on days 5 and 6. Few blastocysts cultured to day 7 ever showed cytoplasmic strings (Figure 3A). The percentage of cytoplasmic strings were similar across all other stages except stage 2 (Figure 3B).

The ICM/TE grades were also referenced to David Gardner as follows: (A) a large number of cells and closely packed; (B) a small number of cells and loosely packed; (C) very few cell numbers (14). The percentages of blastocysts with the presence of strings were significantly less in blastocysts with a Grade C of both ICM and TE than in those with Grades A and B (Figures 3C,D). Thus, the presence of strings was rare in low-quality blastocysts.

### Pregnancy and perinatal outcomes

**Figure 4:**
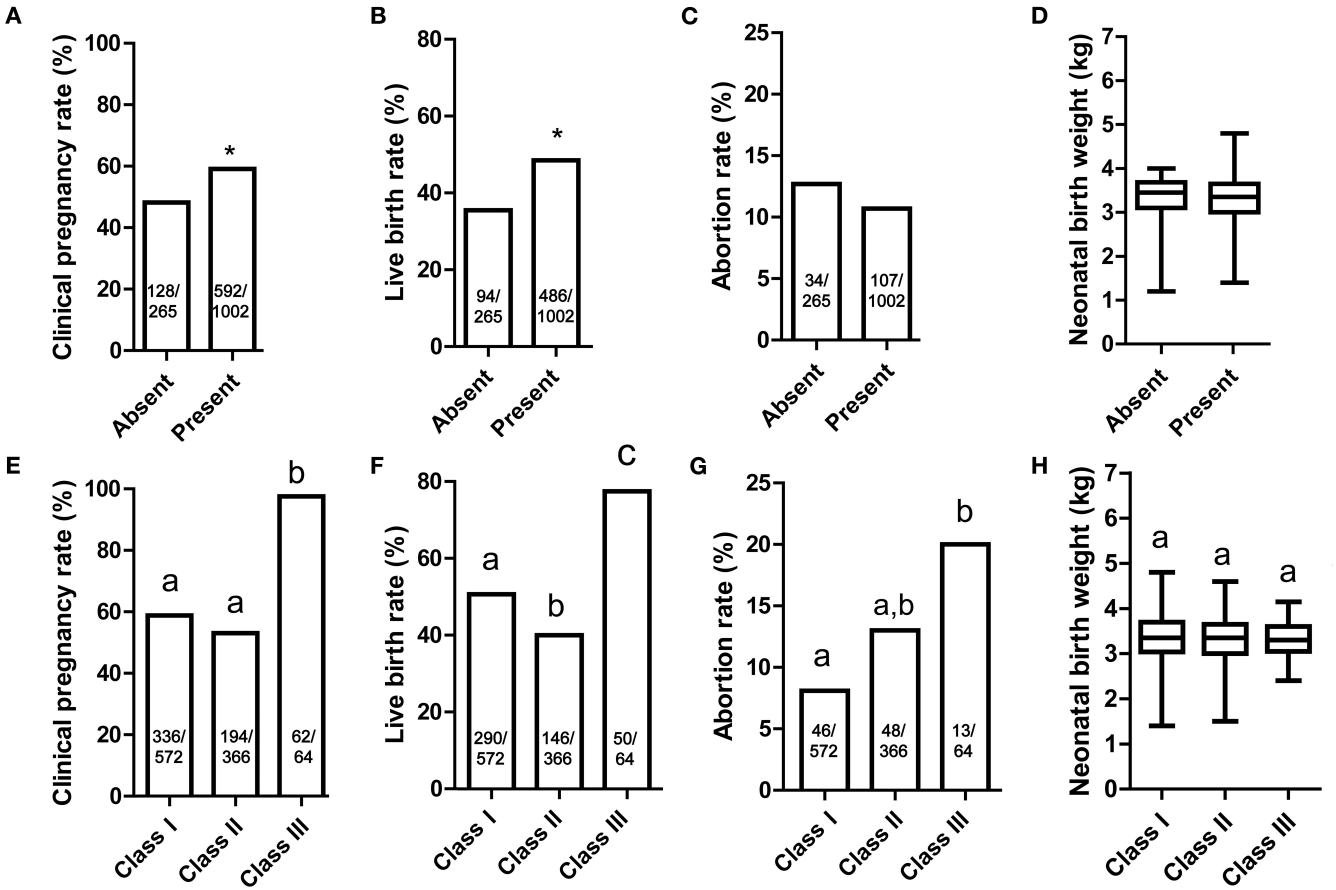
The outcomes of blastocyst with cytoplasmic strings between ICM and mTE cells. **(A)** Clinical pregnancy rate of Absent and Present groups; **(B)** live birth rate of Absent and Present groups; **(C)** abortion rate of Absent and Present groups; **(D)** neonatal birth weight of Absent and Present groups; **(E)** clinical pregnancy rate of Class I, II and III groups; **(F)** live birth rate of Class I, II and III groups; **(G)** abortion rate of Class I, II and III groups; **(H)** neonatal birth weight of Class I, II and III groups. **(A–D)** Absent group was compared with Present group, and significant differences (Chi-square test and Mann-Whitney U test, *P* < 0.05) are indicated by *. **(E–H)** Same superscript lowercase letters indicated no statistical differences in the intergroup comparisons (multiple Chi-square test and Kruskal-Wallis test followed by Dunn's test for multiple comparisons, *P* > 0.05). ICM, inner cell mass; mTE, mural trophectoderm. The sample size was presented on each column.

The main outcome of the study was assessment of the clinical pregnancy rate and live birth rate. 59.1% (592/1002) of blastocysts in the “present” group were implanted, and 48.2% (128/265) of blastocysts were implanted in “absent” group (Figure 4A). Based on this finding, the presence of strings may lead to a better clinical pregnancy rate (*P* = 0.011). Blastocysts in the “present” group also had a higher live birth rate compared with those in the “absent” group (48.5%, 486/1,002 vs. 35.5%, 94/265, *P* = 0.003, Figure 4B). The abortion rate and neonatal birth weight did not differ significantly in two groups (*P* = 0.466 and 0.556, Figures 4C,D).

Among the three classifications of the blastocysts, the clinical pregnancy rate of Class III was much higher than other groups (Figure 4E). The live birth rates for blastocysts in Class III (78.1%, 50/64) was much higher than Class I and II (50.7%, 290/572 and 39.9%, 146/366), respectively (Figure 4F). Although the abortion rate for Class III reached 20.3% (13/64), the sample size was too low to result in a detectable difference (Figure 4G). For perinatal outcome, the neonatal birth weight was statistically similar across all groups, from 3.31 to 3.37 kg (Figure 4H).

## Discussion

The current main criterion for blastocyst selection for transfer is morphological features, such as the grades for ICM and TE based on the Gardner system. However, some essential morphological parameters may be neglected by this system, e.g. cytoplasmic strings between cells. It has been reported by observing the ultrastructure of blastocysts that some TE cells at the polar-mural junction extend cell projections to the surface of the ICM cells through cytoplasmic strings (23). Salas-Vidal and Lomeli (16) reported that filamentous actin was detected in the cytoplasmic extensions or filopodia in mouse blastocysts. These include two types of filopodia: (1) short filopodia that extend both from the ICM and the mural TE into the blastocoel cavity; (2) long, thin filopodia that traverse the blastocoel from the mural TE to a central ICM cell. Short filopodia exist in abundance in the blastocoel, and about 40% of blastocysts could be observed with long filopodia.

Cytoplasmic strings between ICM and mTE cells can commonly be observed at the blastocyst stage with a time lapse system. It had been previously reported that 55–60% of blastocysts were observed to show this phenomenon (24). In the present study, we found an even higher incidence, with 79.08% (1,002/1,267) of transferred blastocysts observed to have cytoplasmic strings between ICM and mTE cells. The reason for the higher observed percentage in our study may be that these were transferred blastocysts, rather than all blastocysts. Additionally, these blastocysts were selected as the most likely to result in clinical pregnancy, so we conclude that the cytoplasmic strings were more visible among high quality blastocysts. Therefore, it is crucial to explore the origin and effect of these strings.

The cytoplasmic strings may be caused by the migration of polar TE cells to mural TE (25). Salas-Vidal and Lomeli (16) reported that the cytoplasmic strings between ICM and mTE cells may suggest cellular activity. Filopodia are dynamic, appearing to extend and retract in coordination with cell division (24). The strings were also verified by immunolocalization of the FGFR2 and ErbB3 receptors to indicate direct communication between the mural TE and ICM cells (16). It has also been reported that cytoplasmic strings could receive the required mitotic signals from the ICM. Other studies found that filopodia contained an abundance of E-cadherin, and determined the cell elongation behaviors required by compaction. When α-catenin and β-catenin were knocked down, filopodia quality was reduced dramatically (24, 26). Therefore, E-cadherin may be the main component of filopodia. These cytoplasmic strings not only exist in culture *in vitro*, but also in the uteruses of patients (27). Cytoplasmic strings between ICM and mTE cells are a common physiological phenomenon that occurs during cell growth. Their presence is conducive to communication between mural TE and the ICM cells, and therefore beneficial to blastocyst development.

In present study, we found that the group of presence of cytoplasmic strings between ICM and mTE cells was more common with a higher grade for ICM and TE. Therefore, we conclude that cytoplasmic strings between mTE and the ICM cells may be an indicator of better-quality blastocysts. This may due to the enhanced cellular communication between ICM and mTE cells. Therefore, further research is required to explore the mechanism of the effect of these strings on the clinical and perinatal outcomes for blastocysts. However, there are also limitations in this study. For example, some blastocysts did not expand completely, resulting in incomplete exposure of cytoplasmic strings. Additionally, the blastocysts may be vitrified before the presence of cytoplasmic strings, and the development of cytoplasmic strings may be blocked by extracellular objects like cumulus cells and embryo fragments.

In order to compare the pregnancy outcomes between blastocysts with cytoplasmic strings present and absent, clinical pregnancy, live birth, and abortion rates were evaluated. Higher clinical pregnancy and live birth rates were observed for blastocysts with observable cytoplasmic strings (*P* = 0.011 and 0.003), but abortion rate was not significantly different (*P* = 0.466). In Thomas Ebner's study, string-positive blastocysts and string-negative blastocysts had similar clinical pregnancy (46.3 vs. 41.2%, *P* > 0.05) and live birth rates (42.7 vs. 30.9%, *P* > 0.05). Though the live birth rate of string-positive blastocysts was much higher than that of string-negative blastocysts, no significant difference was observed. This may due to the number of included cases: 82 cycles in the string-positive group and 68 cases in the string-negative group (17). With an increased sample size, that study may have found similar results to our findings. In our study, abortion rate was 10.65% in the group with strings present and 12.5% in the groups with strings absent (*P* > 0.05). Among the three subgroups with strings present, the abortion rate ranged from 8.08 to 20%. Due to the low incidence of abortions, no significant difference was observed (*P* = 0.466). However, further research should investigate whether the high abortion rate observed in Class III could have caused by a high abundance of strings. For perinatal outcome, the neonatal birth weight was statistically similar across all groups, ranging from 3.31 to 3.37 kg (*P* = 0.556). Thus, although the cytoplasmic strings between ICM and mTE cells could increase the pregnancy and live birth rates, they did not influence the abortion rate and birth weight. This may due to the selective and bidirectional communication between mitotic mTE cells and ICM cells induced by cytoplasmic strings. Though communication between mTE and ICM cells can be accomplished by diffusion of secreted molecules into the fluid, this method may also bring these signals to cells that have already ceased proliferation. Cytoplasmic strings between ICM and mTE cells lead to more effective communication, enable more efficient delivery of substances, and participate in exploration of the extracellular environment. Therefore, our novel findings suggest that cytoplasmic strings between ICM and mTE cells are a positive indicator of increased clinical pregnancy and live birth rates.

## Conclusions

In conclusion, cytoplasmic strings were more commonly observed in good quality blastocysts, and were associated with higher clinical pregnancy and live birth rates. This study provides the first evidence that cytoplasmic strings between ICM and mTE cells can serve as a positive predictor of pregnancy and live birth outcomes in frozen-thawed eSBT cycles. The characterization and functional study of cytoplasmic strings between ICM and mTE cells require further investigation.

## Data availability statement

The raw data supporting the conclusions of this article will be made available by the authors, without undue reservation.

## Author contributions

B-XM was responsible for experimental design, data analysis, and manuscript writing. LY and YT conducted data analysis. BH was responsible for coordinating the study and assembling the time-lapse data. BH and LJ contributed to the analysis of the project. All authors contributed to the article and approved the submitted version.

## Funding

This work was supported by the National Natural Science Foundation of China (81801531 and 82004017).

## Conflict of interest

Author YT was employed by Jiangsu Hengrui Pharmaceuticals Co., Ltd. The remaining authors declare that the research was conducted in the absence of any commercial or financial relationships that could be construed as a potential conflict of interest.

## Publisher's note

All claims expressed in this article are solely those of the authors and do not necessarily represent those of their affiliated organizations, or those of the publisher, the editors and the reviewers. Any product that may be evaluated in this article, or claim that may be made by its manufacturer, is not guaranteed or endorsed by the publisher.
